# Light Management in 2D Perovskite Toward High-Performance Optoelectronic Applications

**DOI:** 10.1007/s40820-024-01643-7

**Published:** 2025-02-06

**Authors:** Kailian Dong, Tao Jiang, Guoyi Chen, Hongsen Cui, Shuxin Wang, Shun Zhou, Chen Wang, Yi Yang, Fang Yao, Chen Tao, Weijun Ke, Guojia Fang

**Affiliations:** 1 Key Lab of Artificial Micro- and Nano-Structures of Ministry of Education of China, School of Physics and Technology, Wuhan University, Wuhan, 430072 People’s Republic of China; 2 School of Electronic and Electrical Engineering, Wuhan Textile University, Wuhan, 430200 People’s Republic of China

**Keywords:** Light management, Dion-Jacobson perovskite, Surface-patterned, Optoelectronic applications, Polarization-sensitive

## Abstract

Two-dimensional Dion-Jacobson (DJ) perovskite has garnered significant attention due to its superior responsivity and operation stability. However, efforts are predominantly focused on discovering new organic spacer to synthesize novel perovskites, while material-form-associated light management, which is crucial for enhancing the photodetector’s efficiency, is largely overlooked. Herein, we introduced surface light management strategy into DJ-type perovskite system by synthesizing surface-patterned BDAPbBr_4_ (BPB, BDA = NH_3_(CH_2_)_4_NH_3_) microplates (MPs) using template-assisted space-confined method, which was further elucidated by theoretical optical simulation. By leveraging surface-patterned MPs to enhance light absorption, the BPB-based photodetectors (PDs) achieved remarkable photoresponse in ultraviolet region, marked by a high on/off ratio (~ 5000), superior responsivity (2.24 A W^−1^), along with large detectivity (~ 10^13^ Jones) and low detection limit (68.7 nW cm^−2^). Additionally, the PDs showcased superior light communication and imaging capabilities even under weak-light illumination. Notably, the anisotropic nature of the surface-patterned MPs conferred excellent polarization sensitivity to the PD. These results represented the first demonstration of BPB perovskite in weak-light communication and imaging, as well as in polarized light detection. Our findings offer valuable insights into enhancing photodetector performance and optoelectronic applications through surface light management strategies.

**Supplementary Information:**

The online version contains supplementary material available at 10.1007/s40820-024-01643-7.

## Highlights

- The light management strategy is introduced into two-dimensional Dion-Jacobson (2D DJ) perovskite and subsequently elucidated both experimentally and theoretically.
- The synthesis of surface-patterned BDAPbBr_4_ microplates with high crystalline quality demonstrates the first reported instance of growing such 2D DJ-type perovskite microplates.
- The optimized device exhibits excellent photodetection performance under UV region. Moreover, this work represents the successful demonstration of BDAPbBr_4_ perovskite for UV weak-light communication, imaging, and polarized light detection.

**Supplementary Information:**

The online version contains supplementary material available at 10.1007/s40820-024-01643-7.

## Graphical abstract

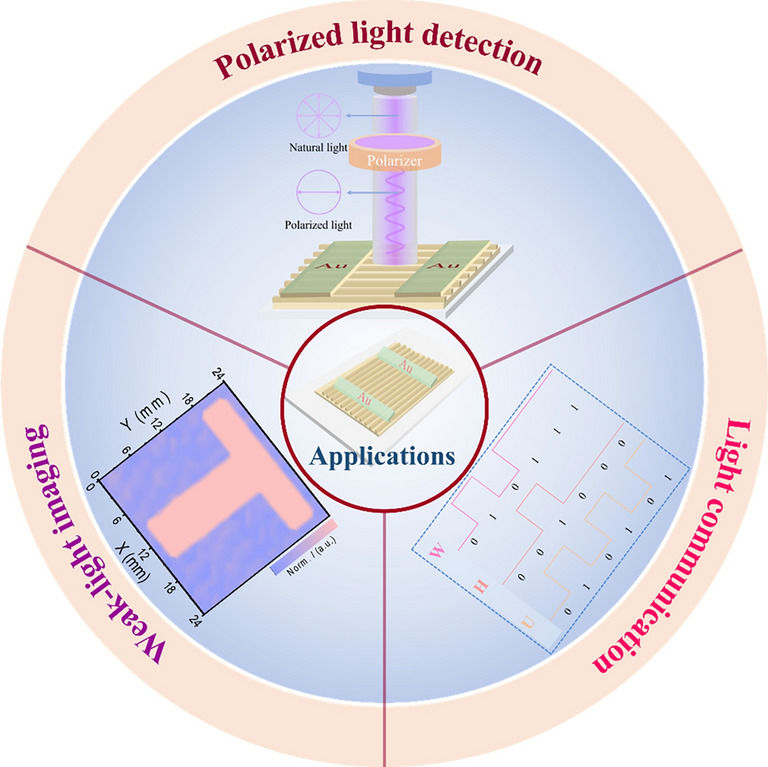

## Introduction

Nowadays, metal halide perovskites have emerged as key candidates for the development of high-performance optoelectronic devices due to their exceptional properties, such as high defect tolerance, large optical absorption coefficient, and high carrier mobility-lifetime product [1–5]. These characteristics make perovskites ideal for a wide range of applications, including solar cells, light-emitting diodes (LEDs), lasers, and photodetectors (PDs) [6–10]. To optimize the performance of perovskite optoelectronic devices, strategies such as interface engineering, compositional engineering, additive engineering, surface passivation, and device architecture optimization [5, 11–14] have been reported. Besides these strategies, significant opportunities to improve efficiency and maximize photon extraction lie in effective light management. Effective light management in perovskite devices involves enhancing light absorption and minimizing reflection losses to ensure maximum conversion of incident light into electrical signals. Strategies such as incorporating nanostructures, using textured surfaces, and integrating photonic crystals have been employed to manipulate the optical path within the device, thereby increasing the interaction between light and the perovskite material [15–17]. For light management in solar cells, resonant structures offer a wave-optics approach to exceed the limitations of light, which have been successfully incorporated in silicon and III-V materials for efficient thin solar cells [18, 19]. Recently, Feng et al. experimentally demonstrated a resonant perovskite solar cell through multiple guide-mode resonances by momentum matching of waveguided modes and free-space light. By utilizing this light management strategy, they achieved an 18-nm band edge extension and 1.5 mA cm^−2^ improvement of the current [20]. Tailoring extrinsic optical properties through scattering structures, micro/nanostructured light outcouples, refractive index matching, optical microcavity effects, and surface plasmon structures is particularly beneficial for perovskite LEDs. These strategies can be adjusted to maximize light extraction and enhance diode efficiency. A common strategy for extracting trapped photons in planar LEDs involves introducing light scattering or outcouple structures [21]. Zhang et al. featured hexagonal arrays of nano-domes serving as both the barrier layer and light outcouple, and a titanium dioxide nanowire array embedded in the anodic alumina membranes (AAM), functioning as optical antennas [22]. They fabricated AAMs with different nanostructure geometries to accommodate perovskite LEDs and discovered nearly double the efficiency of a planar control device. For perovskite PDs, surface light management is essential, especially for perovskite single crystals (SCs), as a significant portion of incident light is typically reflected at the surface of perovskite SCs, leading to insufficient light absorption. Zhang et al. reported a method to prepare a pyramid-structured perovskite MAPbX_3_ (X = I, Br) SCs surface with minimized light reflection and maximized incident light harvesting [16]. After optimizing, the textured SC-based PDs exhibited enhanced responsivity of 321 A W^−1^ and external quantum efficiency (EQE) of 7191%, nearly two times higher than the control device. Recently, Liu et al. introduced an inverted pyramid-shaped structure on the surface of a MAPbBr_3_ SC via in situ growth process, which reduced light reflection and enhanced light absorption [23]. Moreover, surface microstructure engineering through soft imprinting techniques in MAPbBr_3_ and MAPbCl_3_ for reducing light reflection and improving light absorption has been successfully developed to fabricate high-performance PDs [24, 25]. These studies highlight the importance and potential of light management strategies in significantly enhancing the optoelectronic performance of perovskites. However, previous researches have primarily focused on three-dimensional (3D) perovskites, with studies on two-dimensional (2D) perovskites lagging significantly behind.

2D perovskites offer several advantages for high-performance optoelectronic devices fabrication, including superior environment stability, reduced ionic migration, large exciton binding energy and anisotropic charge transport [26–28]. Classified by crystallographic definition, 2D perovskite could be divided into Ruddlesden-Popper (RP) and Dion-Jacobson (DJ) structures [29]. Typically, the main difference is that DJ structures use diamine cations as interlayer spacer, while RP structures use monoamine ones. Generally, the RP-type perovskite with organic–inorganic layers stacked by van der Waals interactions between monoammonium spacers is the most common 2D perovskite candidates for photodetection. However, the large van der Waals gaps between two monoammonium spacers would impede carrier transport and affect the structural stability of RP perovskites. Therefore, DJ-type perovskites offer advantages over RP-type perovskites in terms of lattice stiffness, structural stability, and carrier transport, highlighting the great potential for their use in developing high sensitivity PDs [30–32]. Recently, the impressive performance of DJ-type perovskites in optoelectronic devices has led to a surge in research interest. For example, Shen et al. synthesized centimeter-sized BDAPbI_4_ and explored their charge-transport properties under X-ray excitation [33]. Later, Zhang et al. fabricated planar-type PDs by using BDAPbI_4_ SCs, showcasing excellent response sensitive, including large linear dynamic range (LDR) of 150 dB, high responsivity along with fast response speed and high operation stability [34]. Very recently, we fabricated devices based on micro/centimeter-sized DPAPbBr_4_ SC and investigated their photoelectric properties under UV/X-ray excitation. The optimized PDs showed a high on/off ratio of 4.89 × 10^4^, a large LDR of up to 154 dB, and demonstrated great potential in weak-light imaging and X-ray detection [35]. Nowadays, an increasing number of organic spacers are being discovered and used to synthesize new DJ-type perovskites SCs, e.g., 4AP (4-amidinopyridine) [36], 1,3-BMACH (1,3-bis(aminomethyl)cyclohexane) [37], 3AMPY (3-(aminomethyl)pyridinium) [38], 3-MNPA (3-methylaminopropylamine) [39], DGA (dimethylbiguanide) [40], CYP (1-cyclohexylpiperazine) [41], NMPD (N-methylpropane-1,3-diaminium) [42], and HIS (histammonium) [43]. Most studies focus on finding new organic spacers to synthesize novel DJ-type perovskites, enriching the DJ perovskite family and enhancing our understanding of their optoelectronic properties. However, these perovskites primarily exist with planar-type structures, where a significant portion of incident light is typically reflected at the surface, resulting in insufficient light absorption within the crystal and ultimately leading to reduced performance in PDs. Additionally, the applications of DJ-type perovskites in UV weak-light conditions, such as optical communication, imaging, and polarized light detection, are not yet fully developed or systematically studied. To our knowledge, there are currently no reports on the simultaneous realization of light communication, imaging, and polarized light detection within a single device.

Motived by above consideration, we have successfully introduced light management strategy into 2D DJ-type perovskite for the first time by synthesizing surface-patterned BDAPbBr_4_ (BPB, BDA = NH_3_(CH_2_)_4_NH_3_) MPs using template-assisted space-confined method, which is further elucidated through theoretical optical simulations. A thorough analysis is performed to assess the morphology, crystal structure, and photophysical properties of these MPs. The findings showcase that the surface with well-patterned BPB MPs possesses remarkable attributes, such as high absorption in UV region, excellent crystal quality, and superior moisture and thermal stability. We fabricated detectors with both planar and patterned structures and compared their optoelectronic properties. The results show that the surface-patterned devices exhibit superior performance, which is closely linked to their unique light management strategy. After further optimization, the patterned PDs exhibited outstanding detection capabilities, including a high on/off ratio of ~ 5000, large LDR = 134.13 dB, fast response speed (ms level), and low noise current of 1.4 fA/√Hz. Besides, these PDs showcase an obvious response even under weak-light illumination, specifically achieving a notable detectivity (D^*^) of ~ 10^13^ Jones and responsivity (*R*) of 2.24 A W^−1^ under 68.7 nW cm^−2^ light illumination. These PDs exhibit excellent stability in moisture, thermal conditions, and operational performance. Importantly, these devices demonstrate excellent weak-light communication and imaging capabilities, as well as remarkable polarization sensitivity. This research holds significant importance in introducing light management strategies into 2D perovskites and advancing their optoelectronic applications.

## Experimental Section

### Materials

All chemicals, including 1,4-Butanediamine Dihydrobromide (BDABr_2_, 98%, Aladdin), lead bromide (PbBr_2_, > 98%, Aladdin), and Dimethyl sulfoxide (DMSO, 99.9%, Alfa Aesar), are used as received without any further purification.

### Preparation of the Substrates

The glass substrate with the size of 15 mm × 15 mm was carefully cleaned with deionized water, acetone, and ethanol in the ultrasonic bath for 30 min before use. To fabricate flat and patterned PDMS substrate. A commercial CD with the protective and printing layers are fully peeled off to reveal the clear nanochannels and cleaned with ethanol in the ultrasonic bath. Then, a mixture solution consisting of polydimethylsiloxanes (PDMS) and curing agent were uniformly coated on a piece of CD disk with exposed nanochannels. After heating at 100 °C for 30 min, the patterned PDMS will be obtained by peeling from the CD. For flat PDMS, the CD was replaced by a flat, clean glass to prepare planar PDMS. The remaining procedures align with those previously mentioned.

### Fabrication of the MPs and PDs

BPB MPs were grown by the template-assisted space-confined method. Briefly, BDABr_2_ and PbBr_2_ with a molar ratio of 1:1 were dissolved in DMSO to prepare precursor solution. Then, a few precursors solution was dropped onto a clean glass and covered with patterned or flat PDMS substrates. Then, it was pressed uniformly with a vertical pressure of ~ 10 kPa and then placed in an oven for 5 days at 40 °C. Finally, BPB MPs with patterned or flat surface could be obtained on glass after peeling off the upper PDMS. For the fabrication of planar devices with an Au/BPB MPs/Au structure, the Au electrode was thermally evaporated onto the BPB MPs using a metal mask to create the photodetectors.

### Material and Device Characterizations

The surface and cross-sectional morphology were observed by field emission scanning electron microscope (FE-SEM; JSM 6700F, Japan). The absorption spectrum was measured by UV–vis spectrophotometer (SHIMADZU mini 1280). Atomic force microscopy (AFM) and KPFM images were obtained with a Bruker Dimension Icon XR AFM. X-ray diffraction (XRD) patterns were collected using XRD, D8 FOCUS X-ray diffraction. All the *I*-*V* and *I*-*t* curves were measured by Keysight B2912A Precision Sources/Measure Unit. A 405 nm laser and homogeneous light obtained by a Xenon arc lamp (Newport) and monochromator were used as light source with its intensity determined by a standard Si detector. All the devices were measured in an ambient atmosphere.

### Optical Simulation

The optical simulations were established for studying the optical properties of the surface-patterned or flat structures with periodic boundary conditions in the x-direction. The model was simplified to a two-dimensional case, and Maxwell’s equations were discretized in the time domain using the finite-difference time-domain method to derive the update equations for the electric and magnetic fields. Periodic boundary conditions were applied in the X-direction of the periodic structure, while a plane wave was used to illuminate the structure from above and the wavelength was 405 nm, with a perfectly matched layer implemented below the structure to absorb the incident wave. The optical field distribution of the structure was then obtained through time-domain iterations.

## Results and Discussion

### Surface-Patterned MPs Growth

**Fig. 1:**
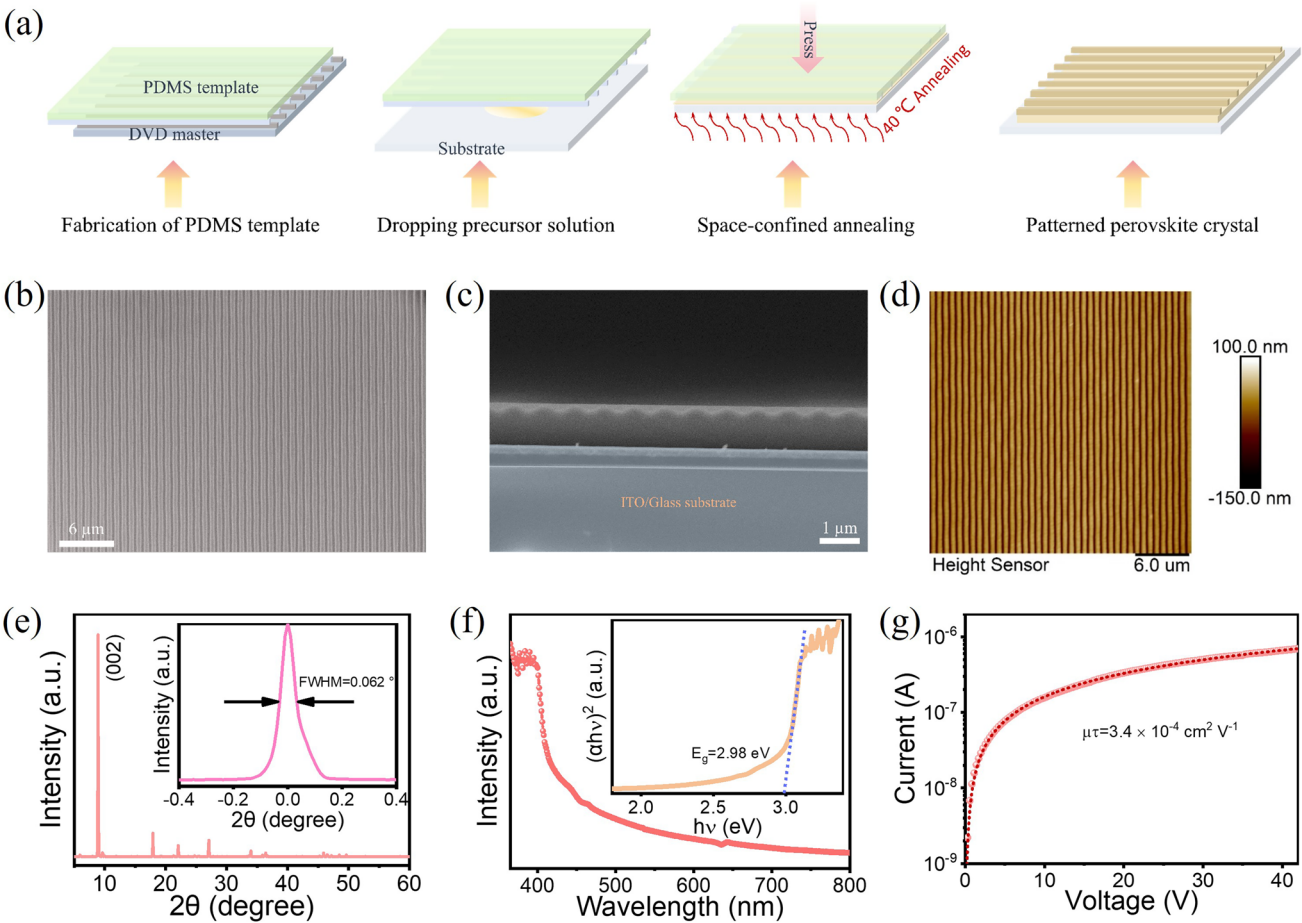
**a** Structural illustration of the template-assisted space-confined crystallization method. **b** Top SEM, **c** cross-sectional SEM, **d** AFM, **e** XRD, and **f** absorption spectrum, and **g** photoconductivity measurement of surface-pattered BPB MPs

Due to the randomness and uncontrollability of perovskite nucleation and crystallization in the precursor solution, it is necessary to use a template constraint to strictly control the crystallization kinetics of the perovskite, thereby obtaining uniformly surface-patterned MPs. In this work, the surface-patterned BPB MPs were synthesized by a simple template-assisted space-confined crystallization method, shown in Fig. 1a. Briefly, we prepared a PDMS template with 1D nanochannels by replicating the grooves of a DVD disk using PDMS at first. Then, the pre-prepared perovskite precursor solution was dropped on a hydrophilic-treated glass substrate and immediately covered it with the PDMS template. Finally, a uniform and constant pressure was applied to the system and it was then transferred to an oven to promote the growth of BPB MPs at 40 °C for 5 days. For control device, a flat PMDS was used to synthesize BPB MPs with flat surface. The crystal structure of BPB was first studied and is depicted in Fig. S1a–c, indicating the typical two-dimensional layered structure of BPB. Typically, BDA^2+^ cation is sandwiched between adjacent inorganic [PbBr_6_]^4−^ polyhedral sheets, creating an alternating inorganic–organic layered structure. Through Hirshfeld surface analysis, the Hirshfeld d_norm_ surface of BPB is shown in Fig. S1d, showcasing the strong N–H···Br hydrogen bonds on both ends between organic cations and inorganic framework. Additionally, to offer a clearer visualization of hydrogen bonding, the hydrogen bonds within BPB are provided in Fig. S1e. Moreover, BPB exhibits a 66.1% of H···Br interaction on the Hirshfeld surface, which is larger than previous reported DPAPbBr_4_ (61.1%), PeA_2_PbBr_4_ (32.6%), HADPbBr_4_ (53.6%), (HIA)_2_AgBiBr_8_ (58.3%), PMA_2_PbBr_4_ (33.4%), (BPEA)_2_PbI_4_ (22.2%), (4ABA)PbI_4_ (59.7%) cations [35, 44–46], indicating the strong hydrogen bond within BPB perovskite (2D fingerprint map is shown in Fig. S1f) [35, 43]. Through the template-assisted space-confined method, surface-patterned BPB MPs could be successfully obtained. We systematically analyzed the morphology and photoelectric properties of BPB MPs. The top scanning electron microscopy (SEM) images of as-grown patterned BPB MPs are shown in Figs. 1b and S2, revealing the successful preparation of the desired patterned perovskite. The SEM indicates that BPB exhibits a nanochannel surface with good uniformity over a large area (Fig. S2a, b) and the crystals show no significant grain boundaries under magnified SEM obversion (Figs. S2c, d). The energy dispersive spectrometer (EDS) indicates that the elements Br and Pb are uniformly distributed on the whole MPs surface (Fig. S3). The uneven spots observed in the EDS mappings are primarily attributed to errors in micro-area analysis, as scanning tiny regions within the sample can lead to irregularities in the elemental distribution. The cross-sectional SEM and its enlarged ones further reveal the well-patterned MPs and no grain boundaries or voids in whole bulk crystal (Figs. 1c and S4). Thanks to the high crystallization quality of MPs, no obvious layering phenomenon is observed in the cross-sectional SEM. Besides, the patterned structure could be confirmed by AFM, in which the height of nanochannel is about 100 µm (Figs. 1d and S5). The above morphological analysis indicates the successful obtained BPB MPs of surface-patterned nanostructures. The XRD patterns of both flat and patterned perovskite reveal that BPB MPs exhibit excellent phase purity with the diffraction peaks matching well with its simulated ones (Figs. 1e and S6). Additionally, the identical orientation observed between flat and patterned perovskites indicates that the arrangement of patterned channels does not influence the crystal growth orientation, which is consistent with previous reports [16, 23]. The disordered crystallization peaks observed in the XRD pattern are attributed to the randomly distributed perovskite film in some region, resulting from the fast and uncontrollable crystallization rate, particularly noticeable at the substrate edges. Moreover, the as-grown MPs display a good crystalline quality with the full-width at half-maximum (FWHM) of (002) plane calculated to be only 0.062° (inset Fig. 1e), comparable to flat ones (Fig. S6b) and some previous reported perovskite SC [47, 48], demonstrating the excellent crystal quality. The optical absorption of BPB MPs is given in Fig. 1f, revealing the high optical absorption at UV region with a clear absorption edge at about 415 nm. Furthermore, the band gap of BPB could be calculated to be 2.98 eV from the corresponding Tauc plot (inset Fig. 1f). To investigate the carrier transport property of BPB MPs, bias-depended photoconductivity measurement has been performed. The carrier mobility-lifetime product (μτ) can be calculated by fitting with Hecht equation [1]:

1 $$I = \frac{{I_{0} \mu \tau V}}{{L^{2} }}\frac{{1 - \exp \left( { - \frac{{L^{2} }}{\mu \tau V}} \right)}}{{1 + \frac{Ls}{{V\mu }}}}$$

where *I*_*0*_, *L*, *V*, *τ*, and s represent the saturated photocurrent, thickness, voltage, the carrier lifetime, and recombination velocity, respectively. As shown in Fig. 1g, the μτ product of surface-patterned MPs is 3.4 × 10^–4^ cm^2^ V^−1^, which is higher than flat ones (Fig. S7), revealing the more efficient carrier separation, transport, and collection. Considering that stability is crucial for future practical applications, we investigated the moisture and thermal stability of BPB. Surprisingly, XRD patterns reveal that BPB MPs showed no significant phase decomposition even after being stored at 70% humidity for 3 months (Fig. S8a). Moreover, the thermal stability of BPB is assessed with thermogravimetric analysis (TGA) (Fig. S8b). There is no detectable mass loss until 310 °C, revealing the high thermal stability of BPB MPs. Above the temperature, it begins to decompose by losing organic portion of BDABr_2_. The above results indicate that the BPB MPs obtained through template-assisted space-confined method exhibit well-patterned surface, excellent structural uniformity, high crystal quality, and superior moisture/thermal stability.

### Performance Comparation Between Surface-Patterned and Flat MPs

**Fig. 2:**
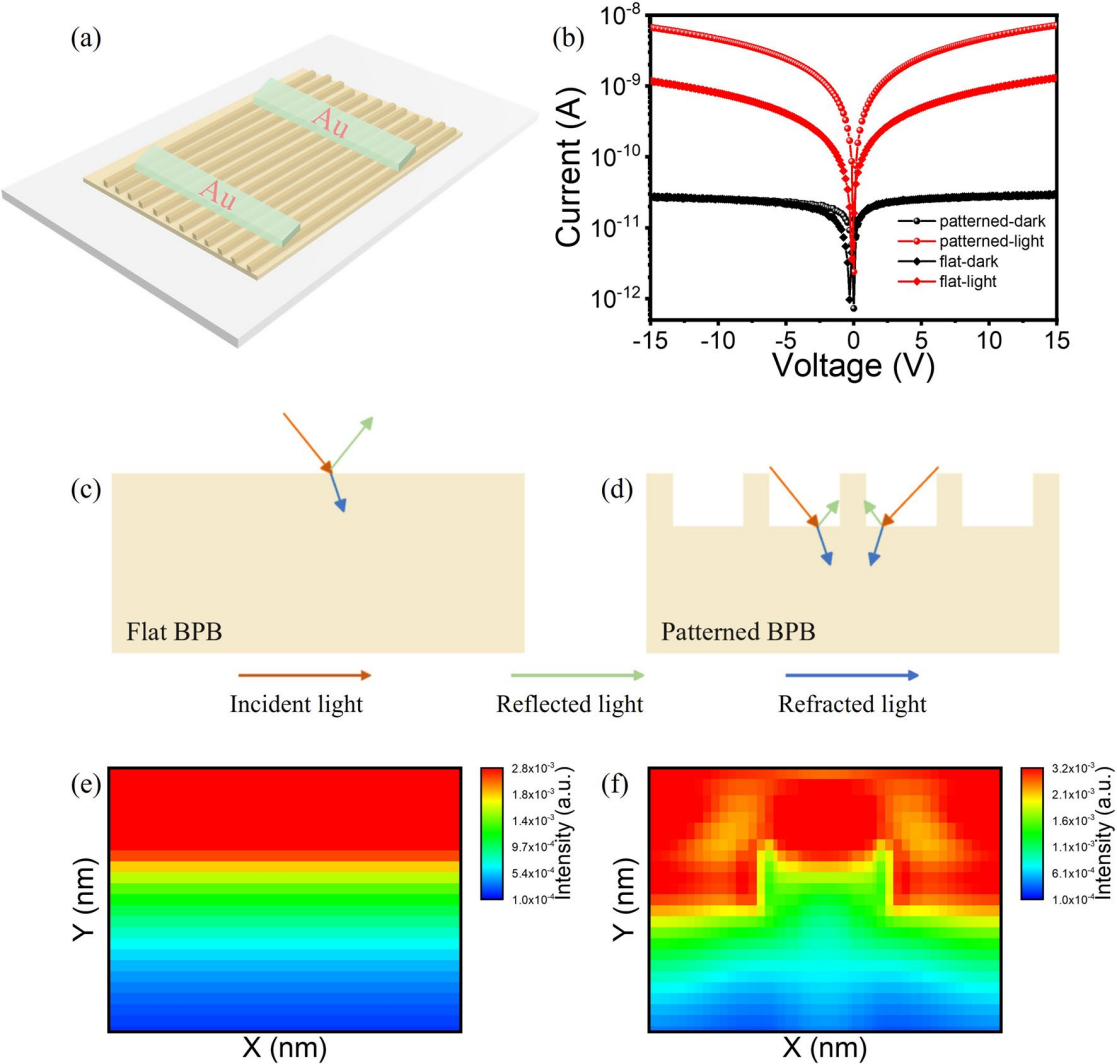
**a** Structure schematic of the surface-patterned PDs. **b** Current comparation of BPB MPs with flat and patterned surface in dark (black line) and under illumination (red line). Illustration of light reflection and FDTD optical simulation under 405 nm light illumination on a flat** c**, **e** and patterned **d**, **g** BPB MPs. (Color figure online)

Given that the high quality of BPB MPs confirmed through above characterization, along with their exceptional absorption and stability, we are motivated to fabricate PDs to further explore their charge-transport properties under UV light excitation. We fabricated Au/BPB/Au devices using a vacuum deposition method with a shadow mask. The schematic illustration and optical microscope image of the patterned PDs are shown in Figs. 2a and S9a, respectively. Similarly, as shown in Fig. S9b, the PDs based on flat-surface MPs are fabricated as control device to verify the feasibility of the light management strategy in surface-patterned structure. The active area of PDs is estimated to be 8.75 × 10^–6^ cm^2^. The current–voltage curves (*I* - *V*) of both PDs were first measured under illumination and dark conditions with the results shown in Fig. 2b. Under dark condition, the suppressed ion migration in 2D perovskites enables both PDs to exhibit exceptional and comparable properties in suppressing leakage current, which is crucial for reducing noise current (*i*_noise_) and detecting weak signals. When illuminated under 405 nm laser, the surface-pattered PD shows a significantly higher photocurrent compared to flat ones. Figure 2c, d illustrates the light propagation processes, comparing a flat surface with normal geometry to a well-patterned surface with light management geometry. Compared to the patterned MPs, a lot of incident light may be reflected at their surface, whereas the well-patterned MPs could trap incident light within their nanochannels, thereby reducing reflection loss. For a more clearly comparation, optical simulations using finite difference time domain (FDTD) for both MPs were performed with the results shown in Fig. 2e, f. The optical field distribution over one optical cycle shows that patterned MPs exhibit a higher optical field, which can enhance optical absorption, promote photocarrier generation, and increase photocurrent. Additionally, the surface-patterned MPs with periodic nanochannels are optically anisotropic, making it advantageous for polarized light detection, as will be detailed later.

### Device Performance of Surface-Patterned MPs-Based PDs

**Fig. 3:**
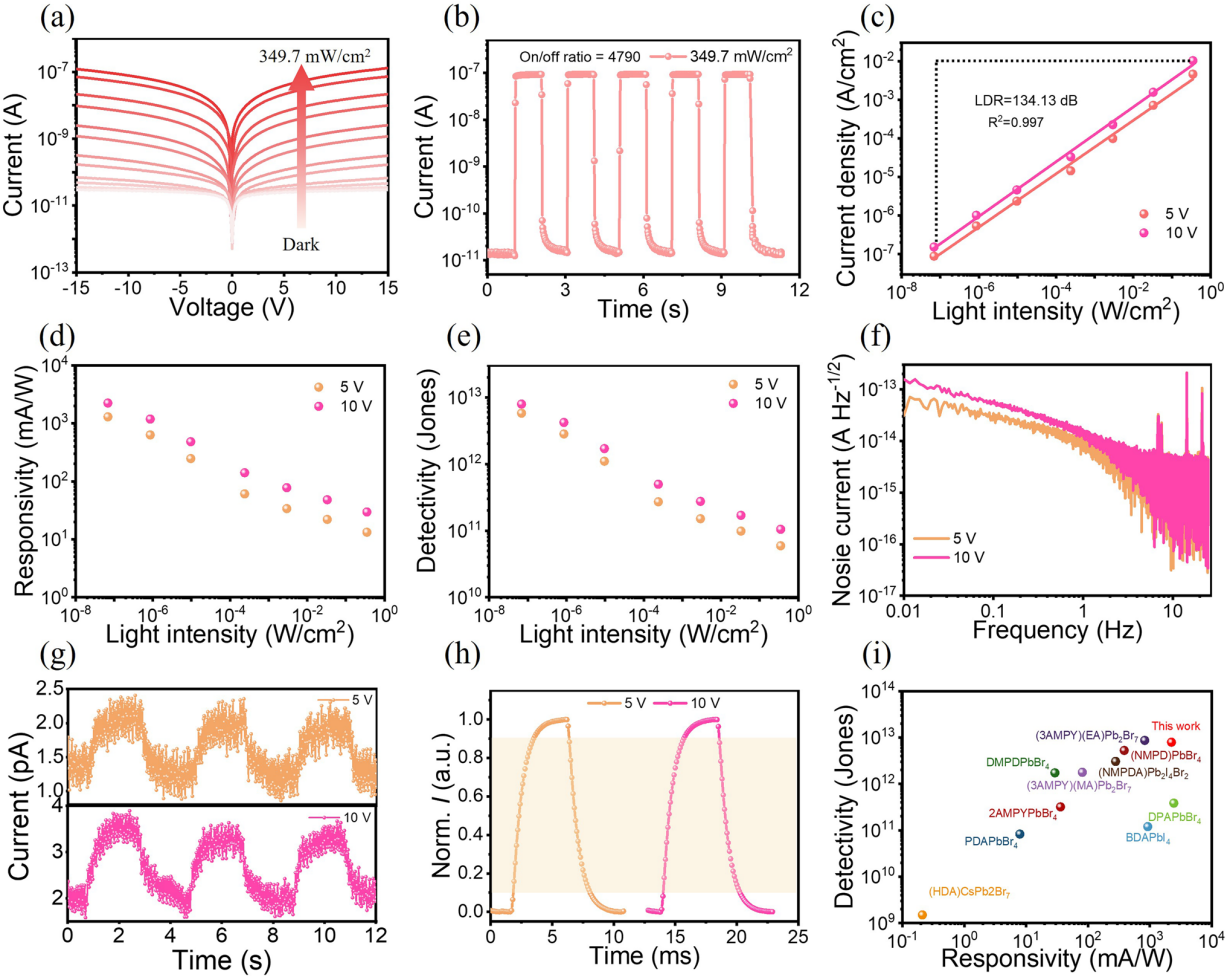
**a** *I*-*V* curves of patterned PDs under 405 nm laser illumination with different light intensities. **b** *I*—*t* curve of patterned PDs under 349.7 mW cm^−2^ 405 nm laser illumination at 10 V bias. **c** *LDR*, **d** *R*, **e** D^*^, **f** noise current, **g** detection limit, **h** response speed of patterned PDs under different bias. **i** *R* and D^*^ comparations of our PD with previous reported DJ-type SC-based perovskite

In-depth theoretical analysis reveals that the surface-patterned PDs offer more significant advantages in photodetection. Subsequently, we conducted a thorough assessment of the optoelectronic performance of surface-patterned PDs. Figure 3a depicts *I*—*V* characteristics of the PD measured in the dark and under 405 nm laser illumination at varying power densities. The results indicate that the photocurrent increases significantly with rising light intensity, resulting from the more generated carries at higher light intensity. Time domain photoresponse was also investigated under different light intensities with the results shown in Figs. 3b and S10. Under 405 nm laser illumination at a power density of 349.7 mW cm^−2^, the PD exhibits a high light current nearly 10^7^ A and low dark current of 1.89 × 10^–11^ A, yielding a high on/off ratio of 4790, which is an essential parameter for optoelectronic devices. Moreover, the PD demonstrates favorable on/off switching characteristics at a wide range of light intensities under 5 and 10 V bias, indicating the high operation stability (Fig. S10a, b). We measured the photo-response of the PD at different light intensities with the results shown in Fig. 3c. It can be seen that the photocurrent of the device shows a linear response from 6.87 × 10^–5^ to 349.7 mW cm^−2^. The photoresponse linearity of a photodetector in a wide illumination range is important for practical applications, which could be described using *LDR*. The *LDR* of the device is calculated to be 134.13 dB corresponding to a ~ 7-magnitude dynamic range, by using following format [47]:

2 $$LDR = 20\log \frac{{P_{{{\text{up}}}} }}{{P_{{{\text{low}}}} }}$$

where P_up_ and P_low_ are the maximum and minimum values of incident light intensity. It is noteworthy that the photoresponse within the measured range does not show significant deviation from linearity at either the minimum or maximum light intensities. Consequently, the *LDR* of the PD is not a saturated value. Based on these photocurrent measurements, the corresponding *R* and D* are calculated using following formats [35, 49]:

3 $$R = \frac{{J_{{\text{p}}} - J_{{\text{d}}} }}{P}$$

4 $$D^{*} = \frac{R\sqrt S }{{\sqrt {2qI_{{{\text{dark}}}} } }}$$

where *J*_p_*, J*_d_*, P, S*, and *I*_dark_ represent the photocurrent density, dark current density, incident light density, effective area, and dark current, respectively.

The *R* and D* are presented in Fig. 3d, e, which show consistent trend that the values increase as the illumination intensity decreases, as more charge recombination is expected under high light intensity, which is a typical characteristic for a photoconductor. The highest *R* and D^*^ are calculated to be 2.24 A W^−1^ and 7.91 × 10^12^ Jones at 68.7 nW cm^−2^ under 10 V bias, which is one of the highest among pervious reported 2D DJ-type perovskite SCs-based PDs (Fig. 3i and Table S1). The noise current is crucial to the detection performance of a PD, particularly for detection limit, and it could be calculated from dark current through Fourier transform. Figure S11 shows the long time dark current of the PD at 5 and 10 V bias. The PD exhibits an ultra-low dark current with an average value of 0.447 pA at 10 V. The ultralow dark current can be attributed to the confinement of carriers within the multiple quantum wells structure of BBP, which is crucial for determining the photodetection performance of the PD [27]. Further, the noise current could be obtained with the results shown in Fig. 3f. The noise current of the PD is approximately 0.1–10 fA/√Hz, and such a low noise current is expected to result in low detection limit. Meanwhile, Fig. 3g illustrates the response of the device under 68.7 nW cm^−2^, demonstrating the excellent weak-light detection performance. To evaluate the response speed of the PD, the photoresponse dynamics are characterized by measuring the rise time (*t*_r_) and fall time (*t*_d_) during a typical photoresponse period, as depicted in Fig. 3h. The *t*_r_ / *t*_d_ are 1.67/1.57 ms at 5 V and 1.69 /1.71 ms at 10 V, along with a -3 dB cutoff frequency of 645 and 590 Hz (Fig. S12), respectively. The fast response speed demonstrates the great potential of the PD for real-time detection and imaging. To access the detection performance of the PD in UV region, the wavelength dependent *R*, D^*^, and *EQE* have been measured, as shown in Fig. S13. The EQE is calculated using the following formula [47]:

5 $$EQE = R\frac{{{\text{hc}}}}{e\lambda }$$

where *h* is Plank’s constant, *c* is the velocity of light, and *k* is the wavelength. The maximum *R*, D^*^, and *EQE* reach 138.2 mA W^−1^, 1.91 × 10^12^ Jones, and 46.7% under 20 V, thereby demonstrating their exceptional sensitivity to UV radiation. Considering the importance of stability in practical applications, we have rigorously assessed the device's photostability, thermal stability, and humidity resistance. As shown in Fig. S14a, the PD shows no obvious current degradation even after continuous 405 nm laser illumination for 100 min. Moreover, under modulated light illumination for ~ 40 min, Fig. S14b indicates that the light and dark currents of the PD are relatively stable, with negligible fluctuations in dark current, underscoring its exceptional operational stability. Surprisingly, the PD exhibits ultra-stable photocurrent even after 2 months exposure to humid air (70% relative humidity) without encapsulation, as shown in Fig. S14c. Additionally, the device exhibits outstanding thermal stability. Figure S14d demonstrates that the current shows no significant change after annealing in ambient conditions for 30 min at 150 °C. The preceding optoelectronic characterization indicates that the surface-patterned BPB detectors exhibit excellent ultraviolet detection capabilities and shows superior stability under light, thermal, and humidity conditions.

### Photoelectric Applications of Surface-Patterned MPs-Based PDs

**Fig. 4:**
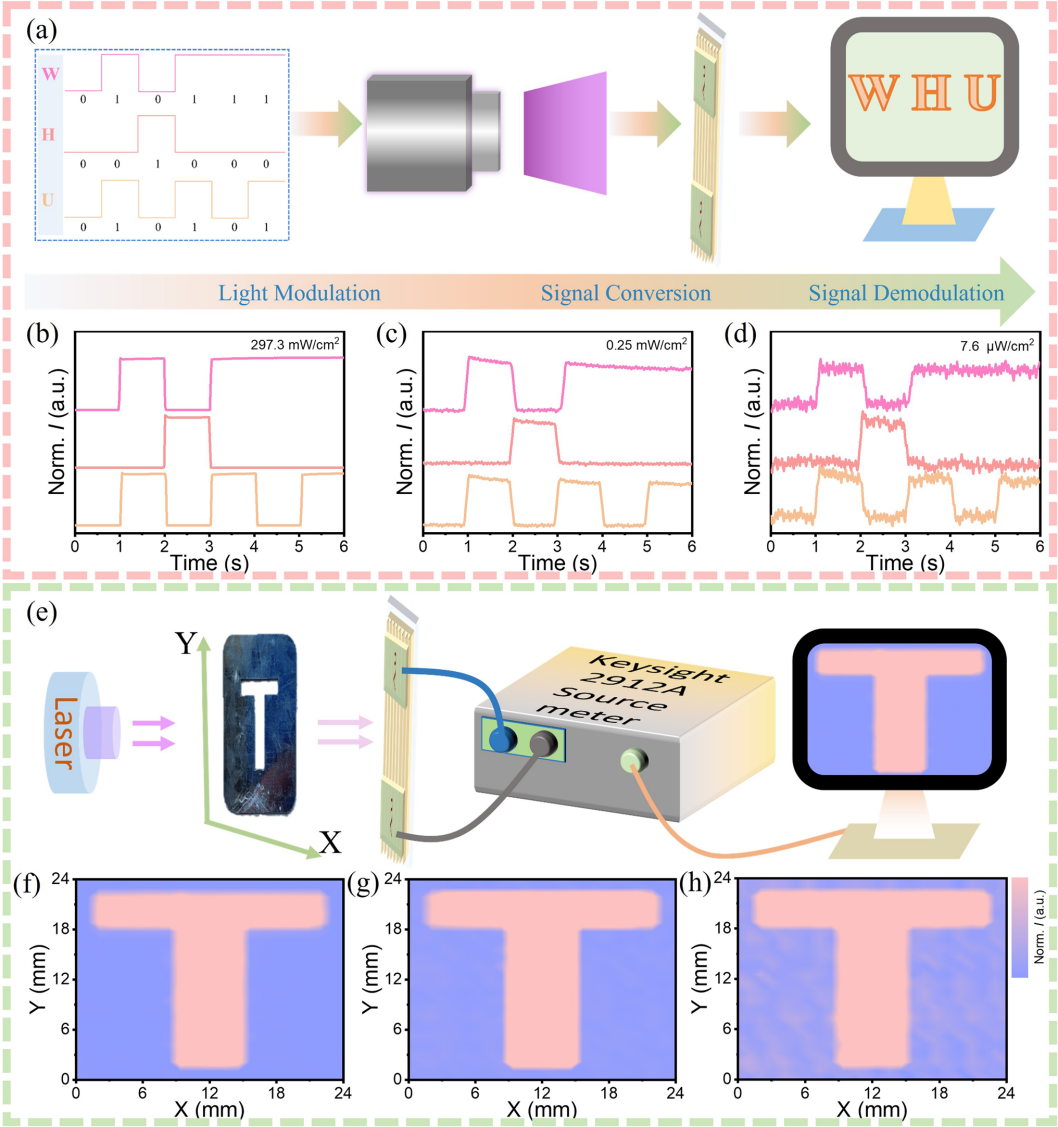
**a** Schematic diagram of the optical communication system based on patterned BPB PD. **b**–**d** Photoresponse characteristics of the patterned BPB PD under modulated 405 nm light. **e** Schematic diagram of the imaging system. The imaging results of patterned BPB PD under 405 nm laser illumination with light intensity of **f** 2.9 mW cm^−2^,** g** 0.25 mW cm^−2^, and** h** 7.6 µW cm^−2^

The aforementioned optoelectronic properties motivate us to explore the practical applications of surface-patterned BPB. Consequently, traditional optical communication and imaging were chosen as the practical application scenarios. In light communication, the process is primarily divided into three components: light modulation, signal conversion, and signal demodulation. In our case, the proof-of-concept demonstration is presented as follows: Firstly, the position numbers of the three letters "WHU" are converted into AMSII codes and modulated onto a 405 nm light source. Typically, W, H, and U are represented as 010111, 001000, and 010101, respectively, where high light intensity corresponds to “1” and low light intensity corresponds to “0.” Then, the signal is subsequently transmitted to the surface-patterned BPB PDs and converted into electrical signals with high and low levels. Finally, the electrical signals are demodulated back into letters to accurately reproduce the complete “WHU” signal. The results of the light communication are depicted in Fig. 4b–d, illustrating that the surface-patterned BPB PDs exhibit exceptional light communication performance even under weak light condition. To demonstrate the imaging capability of the surface-patterned BPB PDs, we designed a single-pixel imaging system, as illustrated in Fig. 4e. For this experiment, a “T”-shaped metal mask, controlled and moved by two stepping motors to allow continuous adjustments along the X- and Y-axes, is positioned between the 405 nm laser and the BPB PD. During imaging, the current of the BPB PD is measured and collected with a source meter (Keysight B2912A) under different light intensities. As illustrated in Fig. 4f, g, the images are exceptionally clear at weak light intensities of 2.9 and 0.25 mW cm^−2^, attributable to the high on/off ratio and rapid response of surface-patterned BPB PD. Importantly, even under 7.6 µW cm^−2^ light illumination, the image exhibits clear distinguishability and is in perfect consistency with the “T”-shaped metal mask. These results demonstrate the significant potential of surface-patterned BPB PDs for practically light communication and imaging even at weak light condition.

### Polarized Light Detection of Surface-Patterned MPs-Based PDs

**Fig. 5:**
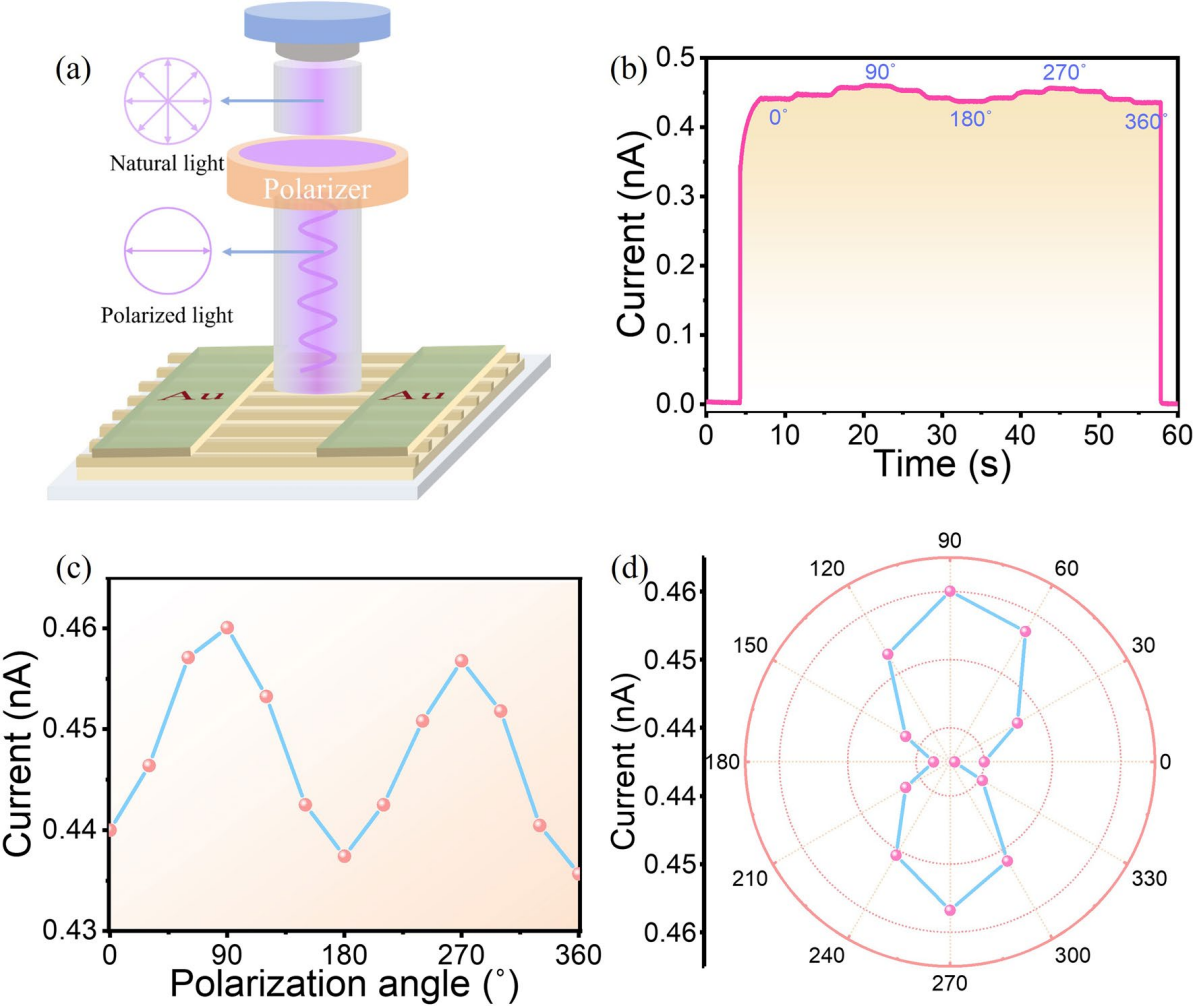
**a** Schematic illustration of the polarized photodetection of patterned BPB PD. **b** *I* - *t* curve at 10 V bias of patterned BPB PD under 365 nm polarized light irradiation as a function of the rotation angle. **c** Photocurrent at 10 V bias of BPB detector as a function of the rotation angle. **d** Polar plot of photocurrent of patterned BPB PD at 10 V bias

Polarized light detection plays a critical role in a range of fields, including polarized light communication, medical imaging, astronomical observation, and scientific research [50–52]. The surface-patterned microplates or nano/microwire arrays usually exhibit optical anisotropy, which significantly enhances their sensitivity to polarized light, and such prospects have been validated in previous reports [25, 53, 54]. The highly anisotropic structure of surface-patterned BPB MPs positions them as promising candidates for polarization-sensitive PDs, and their excellent optoelectronic properties further enhance their ability to detect polarized light. The schematic illustration of polarized photodetection of patterned BPB PD is shown in Fig. 5a. In the experiment, a 365 nm unpolarized light source is employed, followed by a linear polarizer to convert it into linearly polarized light. The angle of the polarizer is then rotated to obtain linearly polarized light at different angles. The device’s response under continuously varying light polarization directions has been evaluated with the result illustrated in Fig. 5b. The polarization angle is varied in 30° increments, and the device exhibits a stable and rapid response to these varying polarization angles. Figure 4b illustrates the photocurrents of the surface-patterned BPB PD as a function of polarization direction. The relationship between the photocurrent and the polarization angle is well-described by a sinusoidal function and the light's polarization direction is 0°; the photocurrent reaches a minimum value and increases to a maximum as the polarization angle increases to 90°. The anisotropy ratio (*I*_max_/*I*_min_) of the device is calculated to be 1.05, which is larger than that of Si-based polarization PDs with the value of 0.5 and is comparable to other perovskite PDs, e.g., (4F-PEA)_2_PbBr_4_ (1.1) [55], MAPbBr_3_ (0.84) [56], BA_2_PbBr_4_ (0.73) [57], CsPbBr_3_ (0.78) [58]. Moreover, the polar plot of the photocurrent for the surface-patterned BPB PD, as depicted in Fig. 5d, demonstrates a strong correlation with the polarization angle. The poor symmetry in the polarization angle-dependent photocurrent (Fig. 5c) and polar plot (Fig. 5d) mainly caused by the inevitable dark current fluctuations, which could be improved through subsequent crystal and structure optimization in future. These findings suggest that the surface-patterned BPB MPs demonstrate significant potential for polarized light detection.

## Conclusion

In conclusion, surface light management strategy is introduced into 2D DJ-type perovskite and elucidated by theoretical optical simulation, for the first time. A facile template-assisted space-confined method is used to synthesize surface-patterned BPB MPs to further elucidate the performance improvement of light management strategy. The PDs based on surface-patterned BPB MPs show improved detection performance than flat ones. After optimization, the surface-patterned BPB PDs exhibit remarkable photoresponse in the UV region, featuring a high on/off ratio of ~ 5000, a high responsivity of 2.24 A W^−1^, along with a large detectivity of ~ 10^13^ Jones, low detection limit of 68.7 nW cm^−2^, and fast response speed. Importantly, those PDs have shown excellent operation stability toward long-term, thermal, and humidity conditions. Furthermore, the PDs have exhibited outstanding light communication and imaging capability even under weak light conditions. Finally, the anisotropic properties of the surface-patterned MPs confer excellent polarization sensitivity to the PD, demonstrating significant potential in polarized light detection. We make a breakthrough by demonstrating that a single device can simultaneously perform light communication, imaging, and polarized light detection. These results represent the first demonstration of BPB perovskite in weak-light communication and imaging, as well as in polarized light detection. This work is significant in introducing light management strategies to perovskites and advancing device performance and optoelectronic applications.

## Supplementary Information

Below is the link to the electronic supplementary material.

Supplementary file1 (DOCX 6666 KB)
