# Supplementary material for: Light Management in 2D Perovskite Toward High-Performance Optoelectronic Applications
Source: Nanomicro Lett. 2025 Feb 6;17:131. doi: 10.1007/s40820-024-01643-7 (PMC11799501; doi:10.1007/s40820-024-01643-7)
Supplement: Supplementary file 1 — Supplementary file1 (DOCX 6666 KB) [file 40820_2024_1643_MOESM1_ESM.docx]

Supporting Information for

**Light-Management in 2D Perovskite towards High-Performance Optoelectronic Applications**

Kailian Dong^1^, Tao Jiang^1^, Guoyi Chen^1^, Hongsen Cui^1^, Shuxin Wang^1^, Shun Zhou^1^, Chen Wang^1^, Yi Yang^1, *^, Fang Yao^2^, Chen Tao ^2^, Weijun Ke^1, *^, Guojia Fang ^1, 2 *^

^1^Key Lab of Artificial Micro- and Nano-Structures of Ministry of Education of China,School of Physics and Technology, Wuhan University, Wuhan 430072, P. R. China

^2^School of Electronic and Electrical Engineering, Wuhan Textile University, Wuhan 430200, P. R. China

Corresponding authors. E-mail: [weijun.ke@whu.edu.cn](mailto:weijun.ke@whu.edu.cn) (Weijun Ke); [gjfang@whu.edu.cn](mailto:gjfang@whu.edu.cn) (Guojia Fang)

**Supplementary Figures and Tables**


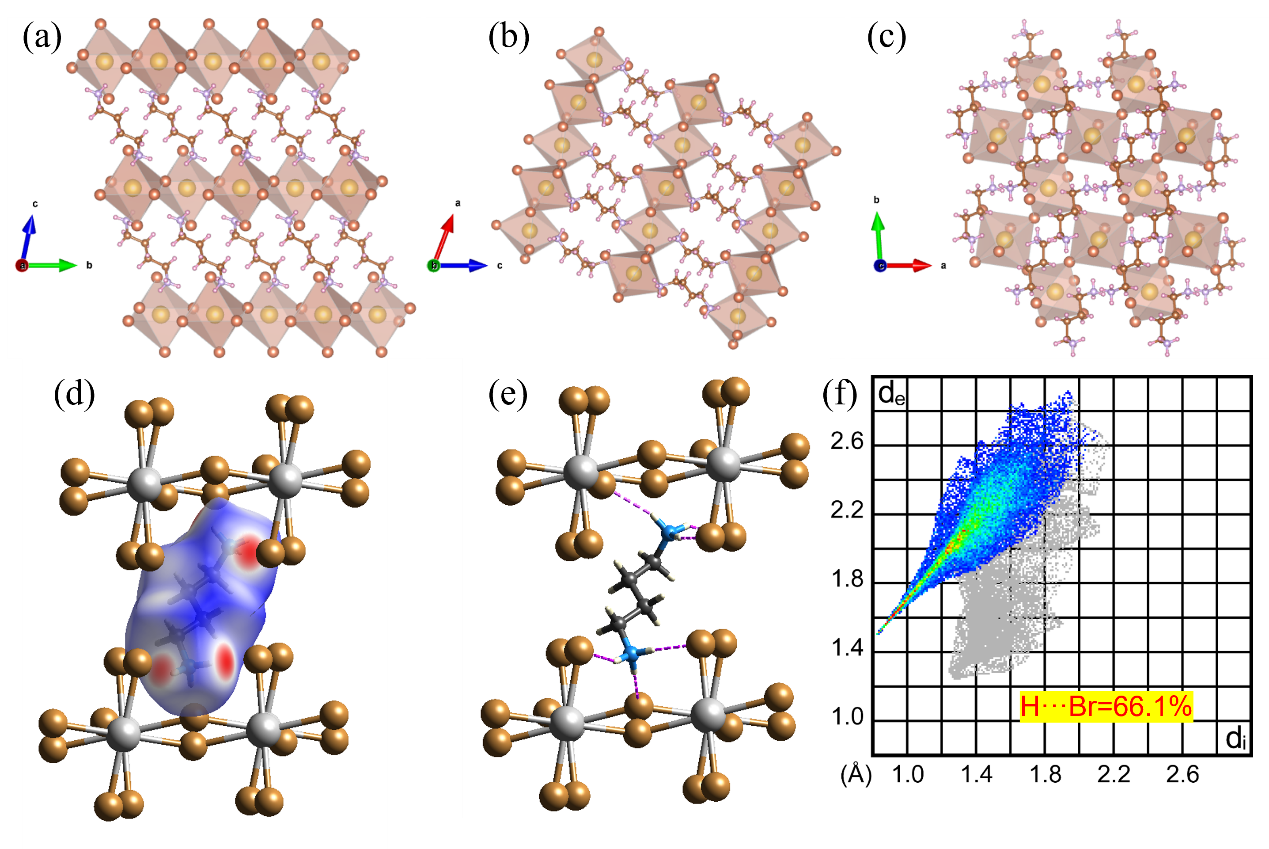


**Fig. S1** **a-c** Schematic diagram of the BPB structure. Hirshfeld d_norm_ surface of BDA^2+^ cation **d**. The hydrogen bonds within BPB **e**. Corresponding 2D fingerprint plots of BPB **f**


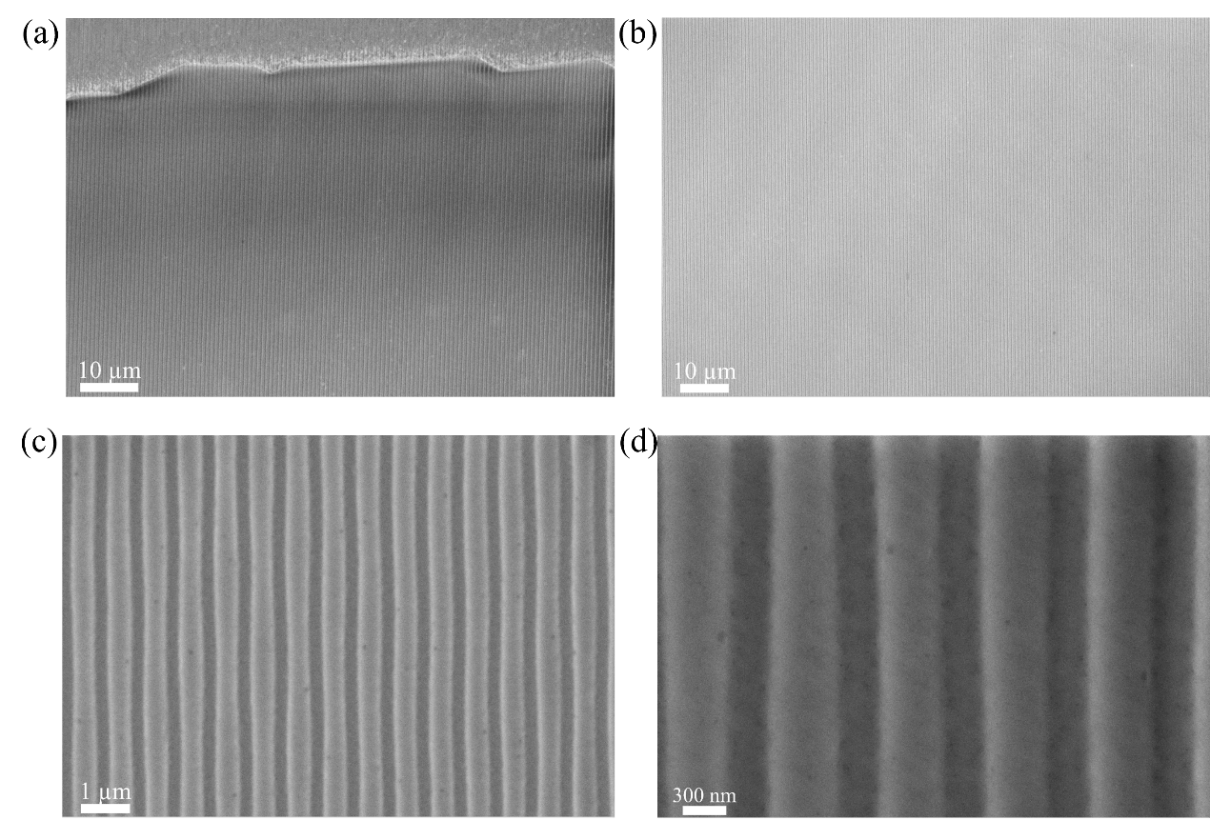


**Fig. S2** Top SEM images of surface-patterned MPs **a-b** and its magnified ones **c-d**


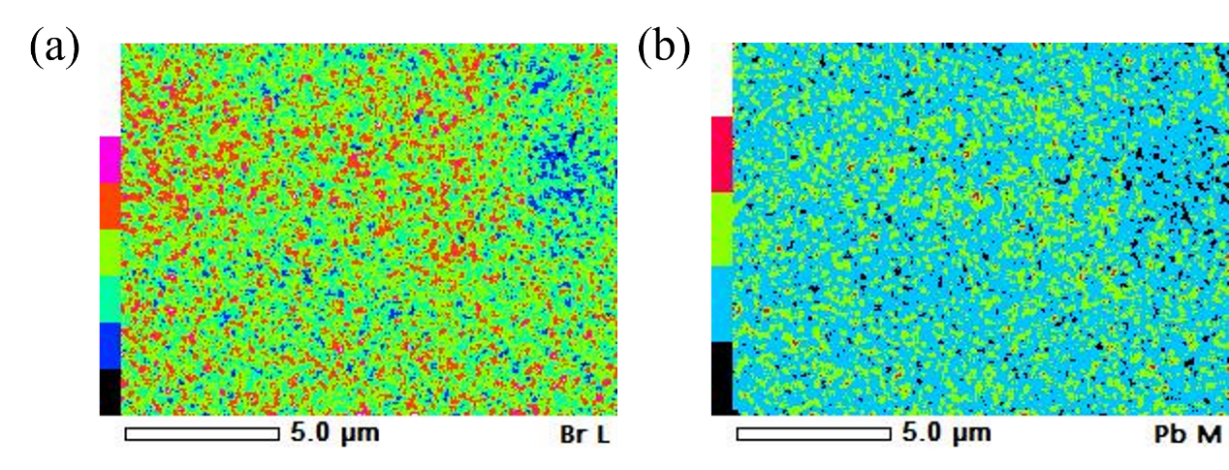


**Fig. S3** EDS mapping of Br **a** and Pb **b** distribution


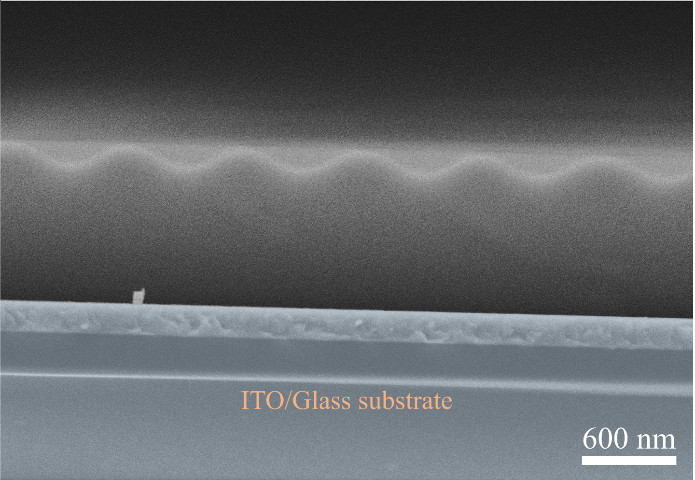


**Fig. S4** Enlarged cross-sectional SEM of patterned BPB MPs


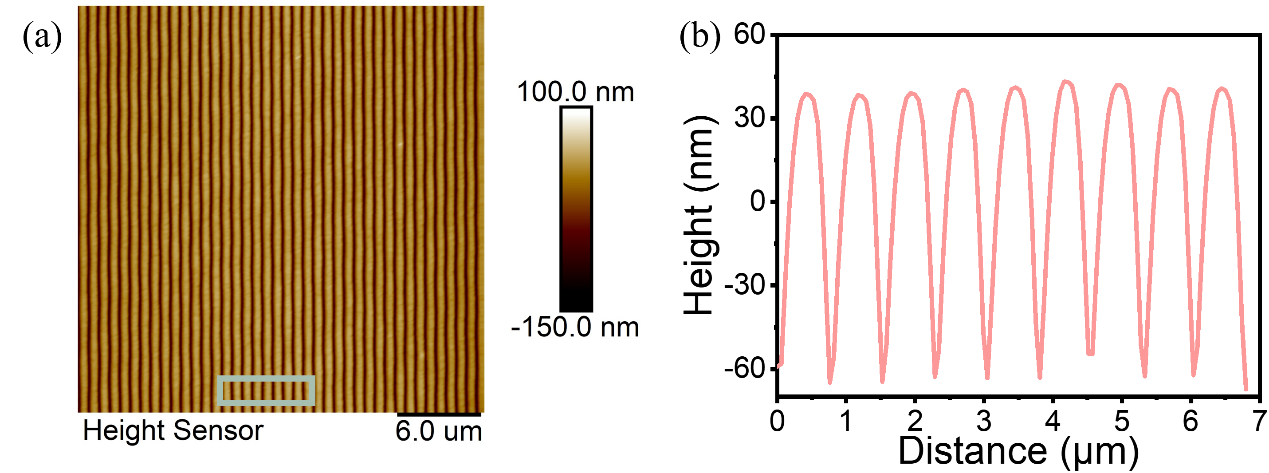


**Fig. S5** AFM and corresponding height of nanochannels


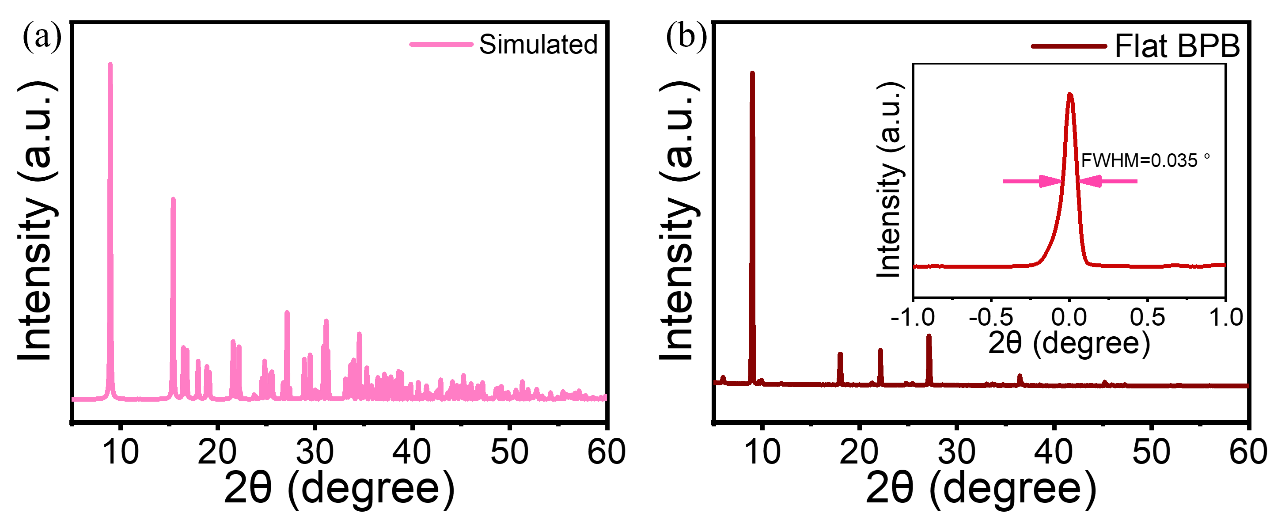


**Fig. S6** Simulated XRD patten of BPB **a**. XRD patten and FWHM of flat BPB **b**


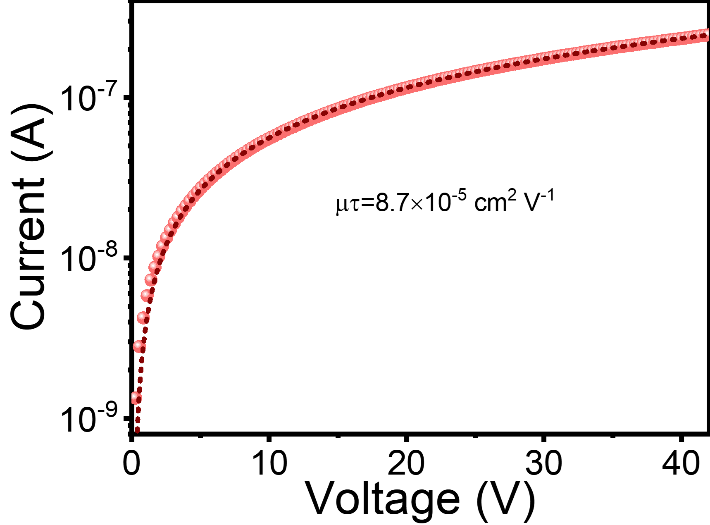


**Fig. S7** Photoconductivity measurement of flat BPB MPs


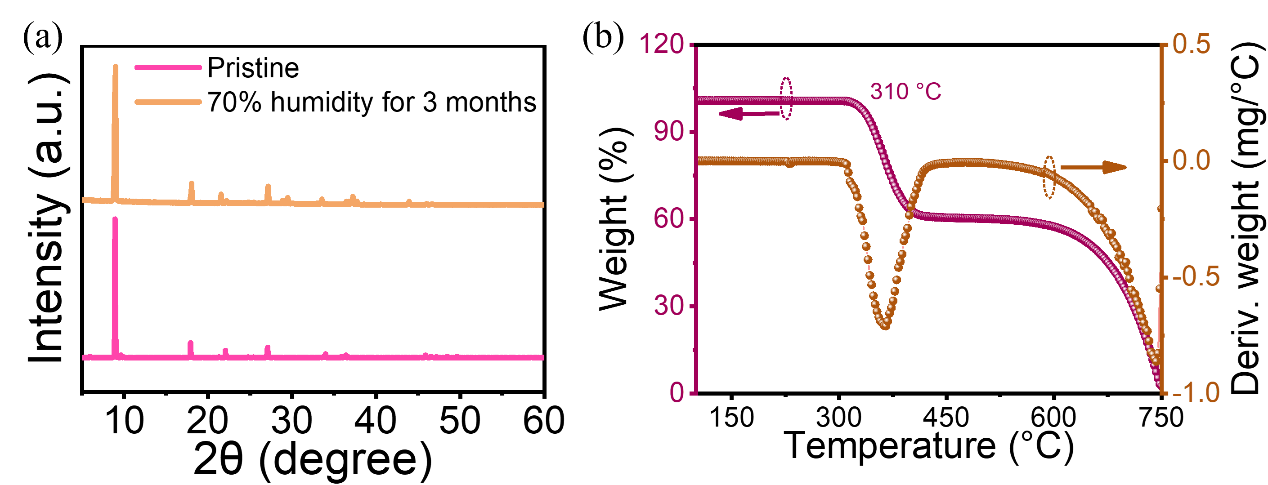


**Fig. S8** XRD patterns of BPB MPs before and after storage under ambient with approximately 70% humidity **a**. TGA curve of the BPB perovskite **b**


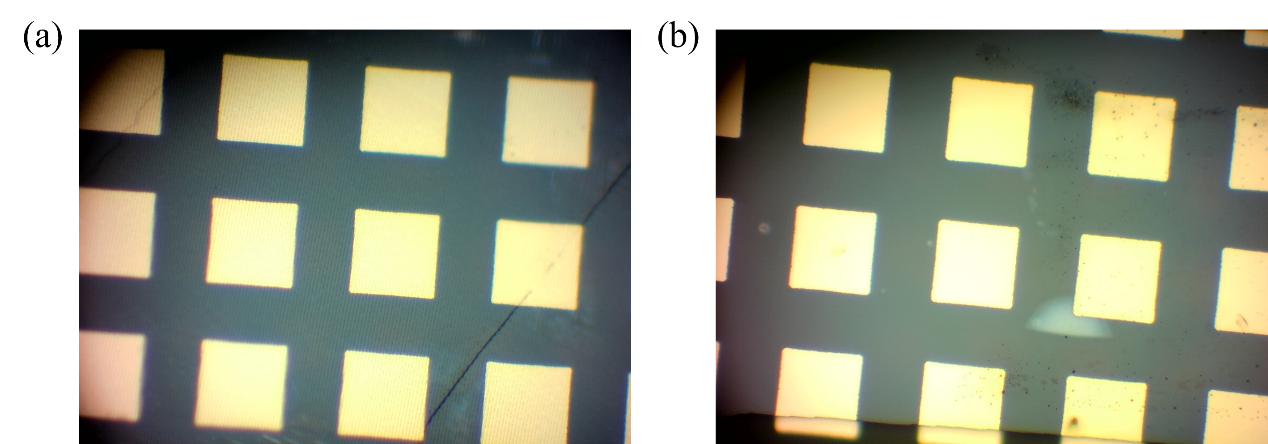


**Fig. S9** Optical microscope images of the patterned PD **a** and flat PD **b**


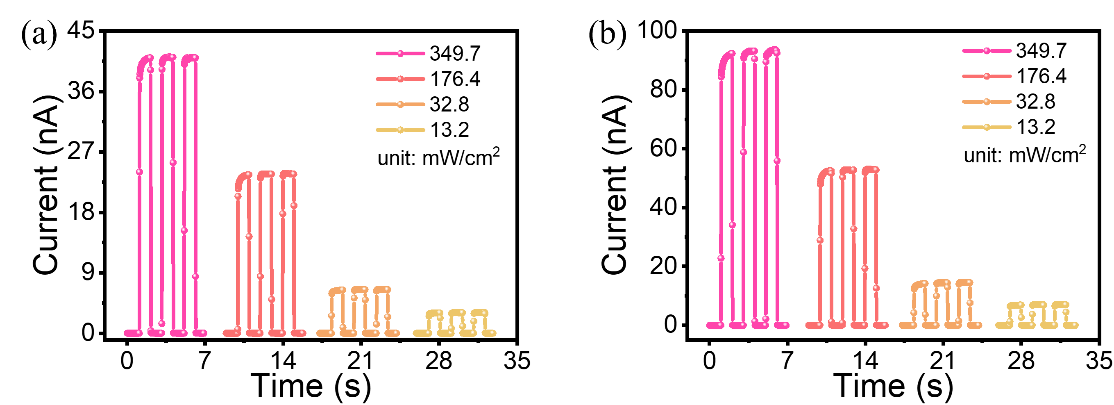


**Fig. S10** Transient photocurrents of the optimized surface-patterned BPB PD under different light intensity at 5V **a** and 10 **b** bias


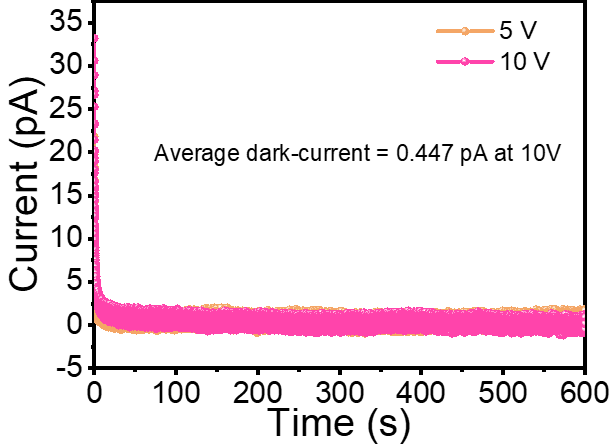


**Fig. S11** Long-term dark current of patterned BPB PD under different biases


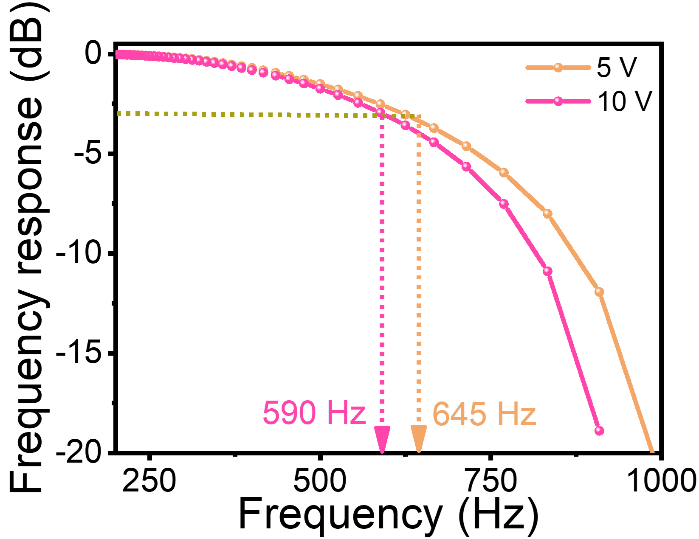


**Fig. S12** -3 dB curves of patterned BPB PD at different biases


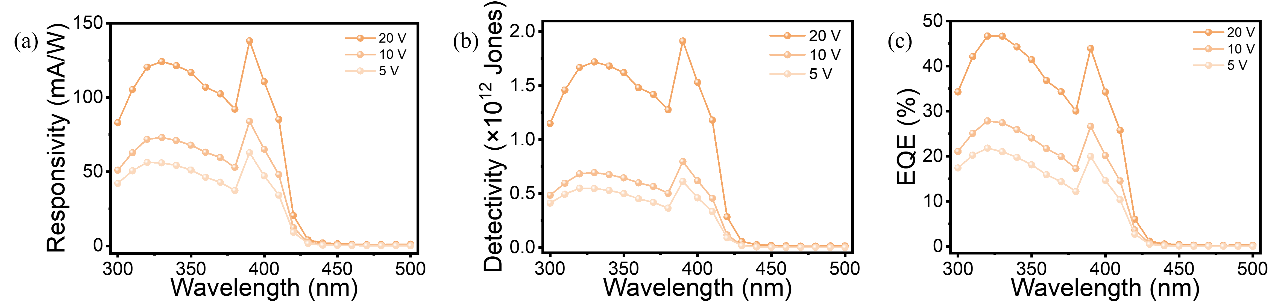


**Fig. S13 a** R, **b** D^*^, and **c** EQE curves of patterned BPB PD as function of wavelength under different voltages


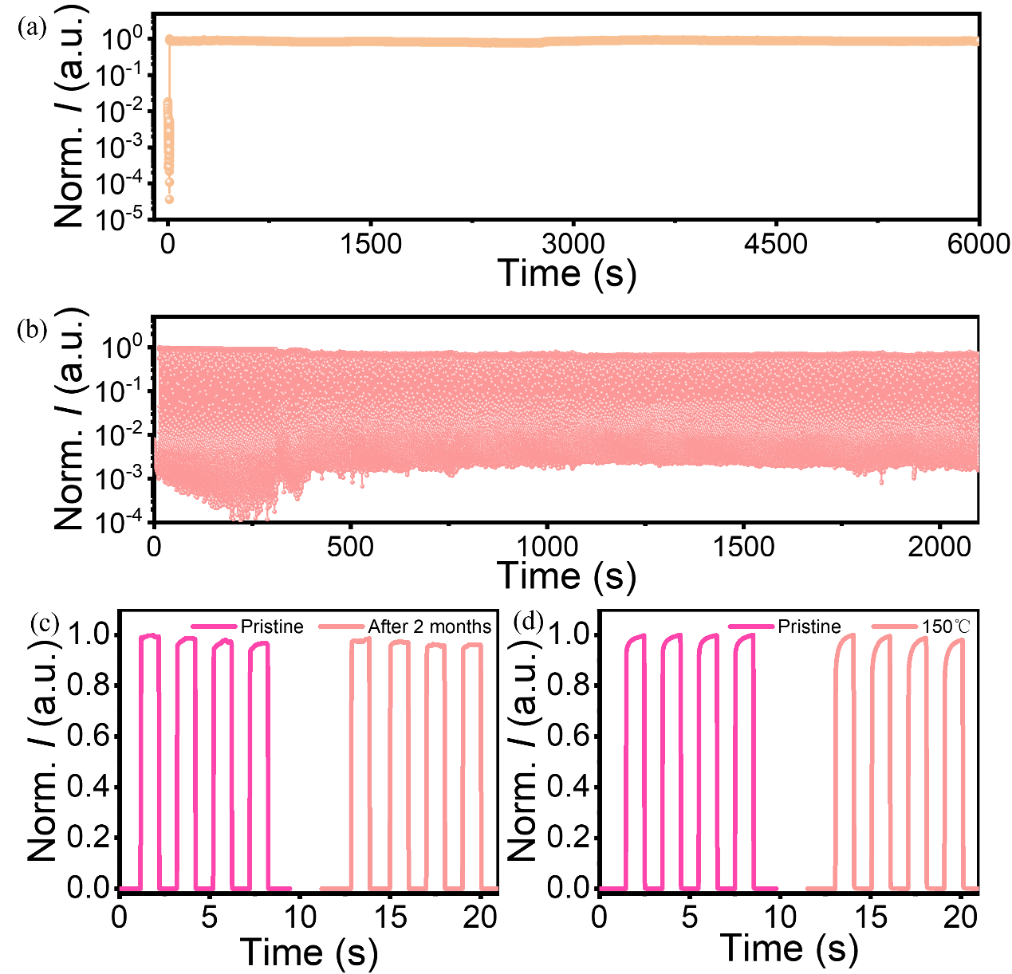


**Fig. S14** The photocurrent response of the patterned BPB PD **a** under modulated light illumination and **b** continuous light illumination. *I* - *t* curves of the patterned BPB PD after storage **c** under approximately 70% relative humidity and **d** post-annealing at 150 ℃

**Table S1** Comparison of our PD with other 2D DJ-type perovskite SCs-based PDs

| Material | Responsivity  (mA/W) | Detectivity  (Jones) | LDR  (dB) | Detection  Limit  (nW/cm^2^) | On/off ratio | Dark current  (A) | Refs. |
| --- | --- | --- | --- | --- | --- | --- | --- |
| BDAPbI_4_ | 927 | 1.2×10^11^ | 150 | 8.8 | 2×10^4^ | 4.2×10^-12^ | [S1] |
| PDAPbBr_4_ | 7.92 | 8.3×10^10^ | - | - | 246 | 5.7×10^-11^ | [S2] |
| DPAPbBr4 | 2444 | 3.85×10^11^ | 154 | 7.1 | 4.89×10^4^ | 4.55×10^-13^ | [S3] |
| DMPDPbBr_4_ | 29.2 | 1.73×10^12^ | - | - | 5.3×10^3^ | 2.69×10^-12^ | [S4] |
| 2AMPYPbBr_4_ | 36 | 3.2×10^11^ | - | - | 10^3^ | 2.87×10^-11^ | [S5] |
| (NMPD)PbBr_4_ | 386 | 5.26×10^12^ | - | - | 9×10^3^ | 3.4×10^-11^ | [S6] |
| (HDA)CsPb_2_Br_7_ | 0.21 | 1.5×10^9^ | - | - | < 4×10^3^ | 2.34×10^-11^ | [S7] |
| (NMPDA)Pb_2_I_4_Br_2_ | 280 | 3.05×10^12^ | - | 1.094×10^4^ | 3.92×10^3^ | 4.2×10^-11^ | [S8] |
| (3AMPY)(MA)Pb_2_Br_7_ | 81.1 | 1.78×10^12^ | - | 70 | 10^3^ | 1.1×10^-10^ | [S9] |
| (3AMPY)(EA)Pb_2_Br_7_ | 827.4 | 8.67×10^12^ | - | ~25 | 2.3×10^4^ | 3.49×10^-11^ | [S10] |
| BDAPbBr_4_ | 2240 | 7.91×10^12^ | 134.13 | 68.7 | 4.79×10^3^ | 4.47×10^-13^ | This work |

**Supplementary References**

1. Y. Zhang, Y. Liu, Z. Xu, Z. Yang, S. Liu, 2D Perovskite Single Crystals with Suppressed Ion Migration for High-Performance Planar-Type Photodetectors. Small **16**, 2003145 (2020). <https://doi.org/10.1002/smll.202003145>
2. W. Yang, J. Hu, J. Chen, Y. Xu, G. Tong, H. Fan, X. Chai, J. Chen, Y. He, Diamine tailored smooth and continuous perovskite single crystal with enhanced photoconductivity. J. Mater. Chem. C **9**, 1303-1309 (2021). <https://doi.org/10.1039/d0tc04746e>
3. K. Dong, X. Yang, F. Yao, H. Cong, H. Zhou, S. Zhou, H. Cui, S. Wang, C. Tao, C. Sun, H. Fu, W. Ke, G. Fang, Spacer Conformation Induced Multiple Hydrogen Bonds in 2D Perovskite toward Highly Efficient Optoelectronic Devices. Adv. Mater. **36**, 2313889 (2024). <https://doi.org/10.1002/adma.202313889>
4. G. Tong, Y. Chen, X. Xiao, J. Tang, B. He, S. Cao, M. Li, Y. He, J. Chen, Asymmetric Diammonium Directed In-Plane Charge Transport Enhancement in Two-Dimensional Lead Bromide Perovskite for Weak-Light Detection. ACS Appl. Mater. Inter. **14**, 53065-53073 (2022). <https://doi.org/10.1021/acsami.2c15570>
5. D. Fu, S. Wu, W. Cao, Z. Chen, X.-M. Zhang, Interlayer cation engineering to regulate the photoelectric properties of lead bromide Dion-Jacobson hybrid perovskites. J. Mater. Chem. C **10**, 9613-9620 (2022). <https://doi.org/10.1039/d2tc01623k>
6. D. Fu, Y. Ma, C.-Y. Su, Z. Chen, D.-W. Fu, Amplification of polarization ratio is observed in monolayer Dion–Jacobson hybrid perovskites. J. Mater. Chem. C **11**, 11492-11499 (2023). <https://doi.org/10.1039/d3tc01926h>
7. T. Yang, Y. Li, S. Han, Z. Xu, Y. Liu, X. Zhang, X. Liu, B. Teng, J. Luo, Z. Sun, Highly-Anisotropic Dion-Jacobson Hybrid Perovskite by Tailoring Diamine into CsPbBr_3_ for Polarization-Sensitive Photodetection. Small **16**, 1907020 (2020). <https://doi.org/10.1002/smll.201907020>
8. B. He, K. Kuang, G. Tong, J. Tang, S. Cao, Z. Yu, M. Li, Y. He, J. Chen, Halide Ordering Enables Superior Charge Transport in 3D (NMPDA)Pb_2_I4Br_2_ Perovskitoid Single Crystal. Small **20**, 2305990 (2023). <https://doi.org/10.1002/smll.202305990>
9. Z. Hou, Y. He, W. Cao, D. Fu, Incorporating an Aromatic Diammonium To Assemble Bilayered Dion-Jacobson Perovskite Crystals for Weak Light Detection. J. Phys. Chem. Lett. **14**, 4304-4312 (2023). <https://doi.org/10.1021/acs.jpclett.3c00755>
10. D. Fu, W. Jia, S. Wu, J. Chang, Z. Chen, J. Luo, Bilayered Dion–Jacobson Hybrid Perovskite Bulk Single Crystals Constructed with Aromatic Diammonium for Ultraviolet–Visible–Near-Infrared Photodetection. Chem. Mater. **35**, 2541-2548 (2023). <https://doi.org/10.1021/acs.chemmater.2c03815>
